# Nuclear Factor-Kappa-B Mediates the Advanced Glycation End Product-Induced Repression of *Slc2a4* Gene Expression in 3T3-L1 Adipocytes

**DOI:** 10.3390/ijms25158242

**Published:** 2024-07-28

**Authors:** Maria Luiza Estimo Michalani, Marisa Passarelli, Ubiratan Fabres Machado

**Affiliations:** 1 Department of Physiology and Biophysics, Institute of Biomedical Sciences, University of São Paulo, São Paulo 05508-000, SP, Brazil; maria.michalani@usp.br; 2 Laboratório de Lípides (LIM-10) do HCFMUSP, Faculdade de Medicina, Universidade de São Paulo, São Paulo 01246-000, SP, Brazil; 3 Programa de Pós-Graduação em Medicina, Universidade Nove de Julho (UNINOVE), São Paulo 01525-000, SP, Brazil

**Keywords:** GLUT4, diabetes mellitus, 3T3L1 adipocyte, NFKB, advanced glycation end products, inflammatory activity

## Abstract

Advanced glycated end products (AGEs) are cytotoxic compounds that are mainly increased in diabetes mellitus (DM), kidney failure, inflammation, and in response to the ingestion of AGE-rich diets. AGEs can also impair glycemic homeostasis by decreasing the expression of the *Slc2a4* (solute carrier family 2 member 4) gene and its GLUT4 (solute carrier family 2, facilitated glucose transporter member 4) protein in muscle. However, the mechanisms underlying AGE’s effect on adipocytes have not been demonstrated yet. This study investigated the effects of AGEs upon *Slc2a4*/GLUT4 expression in 3T3-L1 adipocytes, as well as the potential role of NFKB (nuclear factor NF-kappa-B) activity in the effects observed. Adipocytes were cultured in the presence of control albumin (CA) or advanced glycated albumin (GA) at concentrations of 0.4, 3.6, and 5.4 mg/mL for 24 h or 72 h. *Slc2a4*, *Rela,* and *Nfkb1*mRNAs were measured by RT-qPCR, GLUT4, IKKA/B, and p50/p65 NFKB subunits using Western blotting, and p50/p65 binding into the *Slc2a4* promoter was analyzed by chromatin immunoprecipitation (ChIP) assay. GA at 0.4 mg/mL increased *Slc2a4*/GLUT4 expression after 24 h and 72 h (from 50% to 100%), but at 5.4 mg/mL, *Slc2a4*/GLUT4 expression decreased at 72 h (by 50%). *Rela* and *Nfkb1* expression increased after 24 h at all concentrations, but this effect was not observed at 72 h. Furthermore, 5.4 mg/mL of GA increased the p50/p65 nuclear content and binding into *Slc2a4* at 72 h. In summary, this study reveals AGE-induced and NFKB-mediated repression of *Slc2a4*/GLUT4 expression. This can compromise the adipocyte glucose utilization, contributing not only to the worsening of glycemic control in DM subjects but also the impairment of glycemic homeostasis in non-DM subjects under the high intake of AGE-rich foods.

## 1. Introduction

Advanced glycation end products (AGEs) are generated by the Maillard reaction, leading to the irreversible generation of AGEs [1]. In diabetes mellitus (DM), many biochemical pathways elicited by the increased glucose flow to cells lead to oxidative stress, contributing to intracellular AGE generation [2]. Inflammation and kidney function failure that often accompany DM also contribute to AGE formation. In this sense, the increased plasma levels of AGEs have become a hallmark of uncontrolled DM [3]. However, the body’s AGE pool consists of endogenously generated AGEs and variable amounts of AGEs exogenously acquired from the diet. Western diets are rich in AGEs, mainly from foods processed under long-term, high-heat, and dry cooking conditions [4,5].

In the last decade, AGEs were considered to play an important role in the pathophysiology of diabetes [2,6], participating not only in the development and progression of diabetic complications [7,8] but also in the reinforcement of insulin resistance, eventually worsening glycemic control [3,9]. In addition, in healthy subjects, diet-derived AGEs are major contributors to the body’s AGE pool [10,11], which can achieve levels high enough to induce insulin resistance [12] and participate in the development and progression of several diseases [5,13].

AGEs can contribute to the activation of many cellular stressor mechanisms, including endoplasmic reticulum stress, oxidative stress, and inflammation, all capable of impairing insulin signaling and tissue glucose utilization and contributing to the impairment of glycemic homeostasis [3]. In the main areas responsible for insulin-induced glucose clearance, such as skeletal muscle and adipose tissues, AGEs not only contribute to impairing insulin signaling [12] but are also capable of repressing the expression of the *SLC2A4* (solute carrier family 2 member 4) gene, which codifies the GLUT4 (solute carrier family 2, facilitated glucose transporter member 4) protein [3] as a fundamental player in insulin-stimulated glucose uptake in these tissues [14,15].

Demonstration of the detrimental effects of AGEs in DM is not easy due to the accretion of several other DM pathophysiological mechanisms; however, exposing non-diabetic subjects or biological systems to high levels of AGEs is an appropriate approach to reveal the direct effects of AGEs [16]. Indeed, in 12-week-treated healthy rats with AGE-albumin, insulin resistance was observed, accompanied by the reduced expression of *Slc2a4*/GLUT4 in both adipose and skeletal muscle tissues [3,17]. Additionally, it was demonstrated that increased nuclear factor NF-kappa-B (NFKB) mediated inflammatory activity and played an important role in that effect on skeletal muscle [17]. On the other hand, in adipose tissue, the effects of AGEs have been investigated mainly during the proliferation and/or differentiation of 3T3L1 preadipocytes with the following variable results: reduced cellular proliferation [9] and increased or decreased [18,19,20] cellular differentiation, along with controversial results in insulin-stimulated glucose uptake [20,21], which could be related to different AGE-albumin concentrations or exposition times. Furthermore, some studies have related these AGE effects to oxidative stress [9,18] or NFKB [19] pathway activation. Despite these reports, little is known about the direct effects of AGEs upon the regulation of *Slc2a4*/GLUT4 expression in adipose tissue, especially concerning differentiated adipocytes and the NFKB regulation of its target gene, *Slc2a4*.

Considering this, the present study investigates the effects of advanced glycated albumin (GA) on the regulation of *Slc2a4*/GLUT4 expression in differentiated 3T3-L1 adipocytes, as well as the potential role of NFKB-mediated activity in the observed effects.

## 2. Results

Initially, we investigated if GA could compromise adipocyte viability compared to CA under the same conditions. Cellular viability, analyzed by the MTT method, was unaltered among the groups (Supplementary Figure S1).

### 2.1. Advanced Glycated Albumin Modulates Slc2a4 mRNA and GLUT4 Protein Expression in 3T3-L1 Adipocytes in a Time- and Concentration-Dependent Way

**Figure 1:**
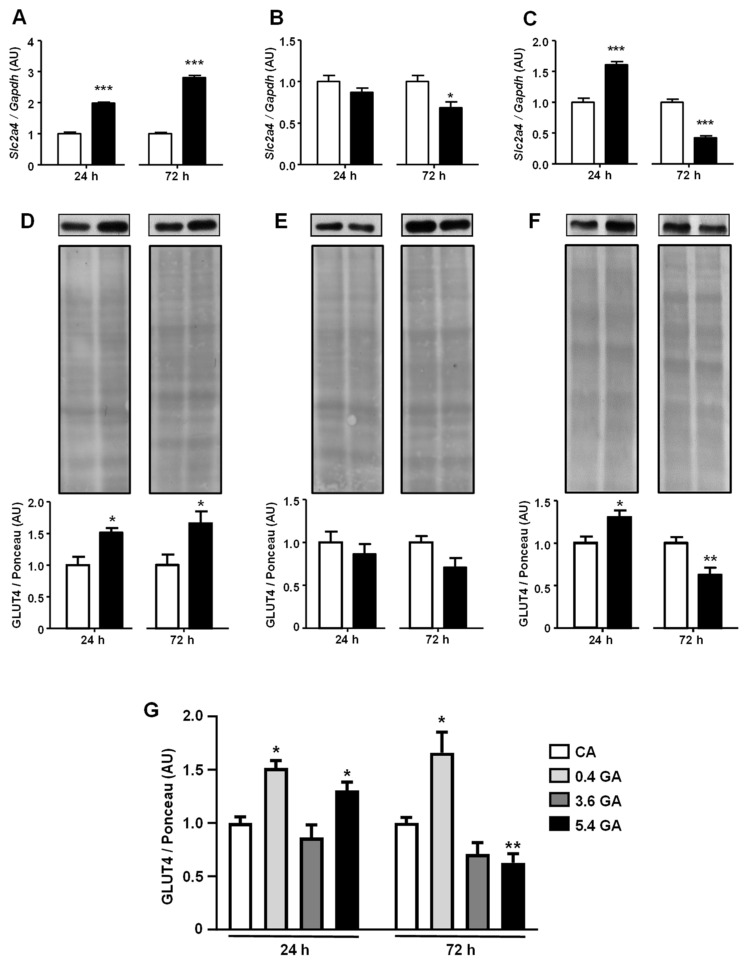
Advanced glycated albumin modulates *Slc2a4* mRNA and GLUT4 protein expression in 3T3-L1 adipocytes in a time- and concentration-dependent way. Adipocytes were cultured for 24 h or 72 h in the presence of control albumin (CA; white bars) or advanced glycated albumin (GA; black bars) at the concentrations of 0.4 (**A**,**D**), 3.6 (**B**,**E**), or 5.4 (**C**,**F**) mg/mL. *Slc2a4* mRNA (**A**–**C**) was quantified by RT-qPCR, using the *Gapdh* mRNA as a loading control; GLUT4 protein (**D**–**F**) was quantified by Western blotting, using the Ponceau-stained membrane as a loading control. The results were normalized by considering the mean of CA values as 1.0 in each concentration/time. In (**D**–**F**), representative blots of GLUT4 are shown at the top of the panel with the respective Ponceau-stained membrane. From (**A**–**F**), data are expressed as the means ± SEM of 4 to 7 samples (from at least 4 different culture plates) and were compared using Student’s unpaired *t*-test (* *p* < 0.05, ** *p* < 0.01, and *** *p* < 0.001 vs. CA). In (**G**), data from all CA concentrations at each time-point (which did not vary) were pooled and thus compared to GA data by two-way ANOVA (time *p* < 0.05; treatment *p* < 0.01; interaction *p* < 0.01), followed by Student’s unpaired *t*-test (* *p* < 0.05 and ** *p* < 0.01 vs. respective CA group). AU, arbitrary units; GLUT4**,** solute carrier family 2, facilitated glucose transporter member 4; *Gapdh*, glyceraldehyde-3-phosphate dehydrogenase; and *Slc2a4*, solute carrier family 2 member 4.

At a 0.4 mg/mL concentration, GA induced a clear increase in both *Slc2a4* mRNA (Figure 1A, *p* < 0.001) and GLUT4 protein (Figure 1D, *p* < 0.05) after 24 h and 72 h. In contrast, 3.6 mg/mL GA induced a tendency for reduction in mRNA (Figure 1B) and protein (Figure 1E), which was significant (*p* < 0.05) only for mRNA after 72 h. However, at 5.4 mg/mL and after 24 h, GA increased the *Slc2a4* mRNA (*p* < 0.001) and GLUT4 protein (*p* < 0.05) again, whereas, after 72 h, GA repressed (by ~50%) the expression of *Slc2a4* mRNA (Figure 1C, *p* < 0.001) and protein (*p* < 0.01).

Considering a certain difficulty in comparing the results analyzed at different set times, a new analysis was performed (Figure 1G) in which all data of GLUT4 were normalized by the mean 24 h CA treatment (set to 1.0), and statistically compared by two-way ANOVA. The analysis revealed there are significant effects of time (*p* < 0.05) and treatment (*p* < 0.01), as well as an interaction between the variables (*p* < 0.01). In addition, the analysis confirmed that 0.4 mg/mL of GA increased (*p* < 0.05) the GLUT4 protein at both 24 h and 72 h, but 5.4 mg/mL displayed a hormetic effect of increasing (*p* < 0.05) at 24 h and decreasing (*p* < 0.01) at 72 h. Images of the original experiments from which the representative images were selected showing the entire Ponceau-stained membrane and the immunoblotted membrane are presented as Supplementary Figures S2–S6.

### 2.2. Advanced Glycated Albumin Increases the Expression of Genes of NFKB Subunits p65 and p50

**Figure 2:**
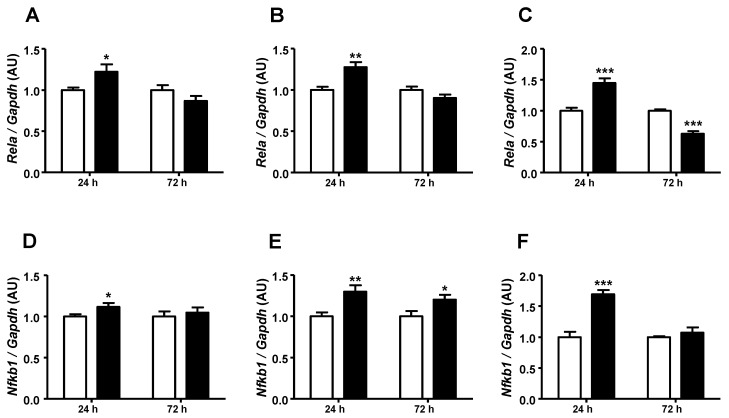
Advanced glycated albumin increases the expression of genes related to proinflammatory activity. Adipocytes were cultured for 24 h or 72 h in the presence of control albumin (CA; white bars) or advanced glycated albumin (GA; black bars) at the concentrations of 0.4 (**A**,**D**), 3.6 (**B**,**E**), or 5.4 (**C**,**F**) mg/mL. *Rela* (**A**–**C**) and *NFKb1* (**D**–**F**) mRNAs were quantified by RT-qPCR, using the *Gapdh* mRNA as a loading control. The results were normalized considering the mean of CA values as 1.0 in each concentration/time. Data are expressed as means ± SEM of 4 to 7 samples (from at least 4 different culture plates) and were compared by Student’s unpaired *t*-test (* *p* < 0.05, ** *p* < 0.01, and *** *p* < 0.001 vs. CA). AU, arbitrary units; *Gapdh*, glyceraldehyde-3-phosphate dehydrogenase; *Nfkb1*, nuclear factor kappa B subunit 1; *Rela*, RELA proto-oncogene; and NF-kB subunit.

To investigate the participation of the inflammatory pathway in the GA-induced regulation of *Slc2a4*/GLUT4 expression, the gene expressions of *Rela* and *Nfkb1* were analyzed. After 24 h, GA treatment increased (*p* < 0.05 to *p* < 0.001) the expression of both *Rela* and *Nfkb1* mRNAs at all concentrations (Figure 2A–F). However, after 72 h, GA showed a preferentially unchanged effect but with a significant (*p* < 0.05) increase in *Nfkb1* at 3.6 mg/dL (Figure 2E) and a decrease in *Rela* (*p* < 0.001) at 5.4 mg/mL (Figure 2C).

### 2.3. Advanced Glycated Albumin Does Not Modulate the Activity of the IKKs at 5.4 mg/dL Concentration

**Figure 3:**
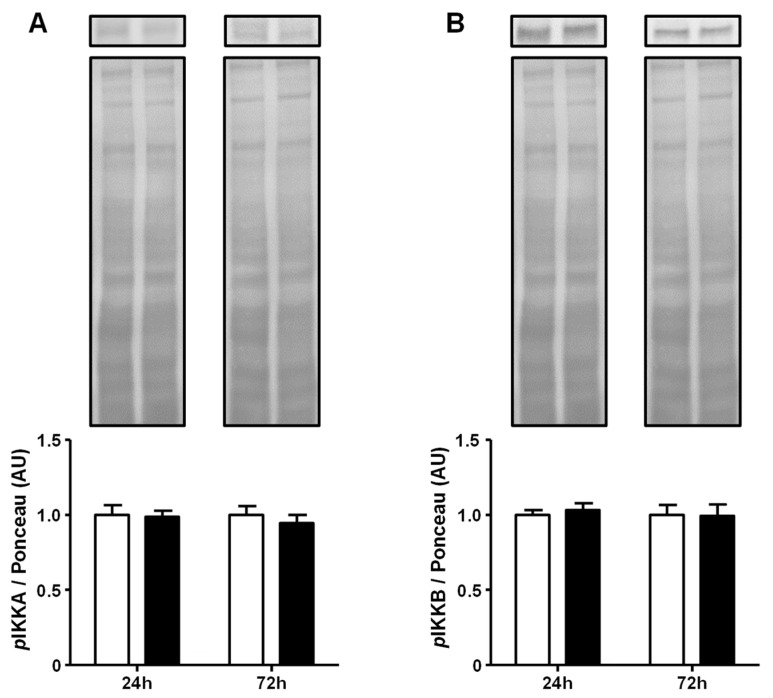
Advanced glycated albumin does not modulate the activity of the IKKs at a 5.4 mg/dL concentration. Adipocytes were cultured for 24 h or 72 h in the presence of control albumin (CA; white bars) or advanced glycated albumin (GA; black bars) at a concentration of 5.4 mg/mL. The phosphorylation (*p*) grade of the catalytic subunits IKKA (**A**) and IKKB (**B**) was analyzed by Western blotting, using the Ponceau-stained membrane as a loading control in a total cellular protein fraction. Representative blots of *p*IKKA and *p*IKKB are shown at the top of the panel with the respective Ponceau-stained membrane. Results were normalized considering the mean of CA values as 1.0 at each time-point. Data are expressed as the means ± SEM of 4 to 6 samples (from at least four independent culture plates). No significant differences were observed. *p*IKKA, phosphorylated inhibitor of nuclear factor kappa-B kinase subunit alpha; *p*IKKB, phosphorylated inhibitor of nuclear factor kappa-B kinase subunit beta.

Aiming to explore the GA-induced activation of the NFKB pathway, the upstream step of the phosphorylation of the inhibitor of nuclear factor kappa-B kinase subunits alpha (IKKA) and beta (IKKB) was evaluated only at a 5.4 mg/mL concentration. GA did not increase the phosphorylation of IKKA and IKKB, neither after 24 h nor after 72 h of treatment (Figure 3). Original experiments from which the representative images were selected showing the entire Ponceau-stained membrane and the immunoblotted membrane are presented in Supplementary Figure S7.

### 2.4. Advanced Glycated Albumin Increases Nuclear Content of Nuclear Factor NF-kappa-B at 5.4 mg/dL Concentration

**Figure 4:**
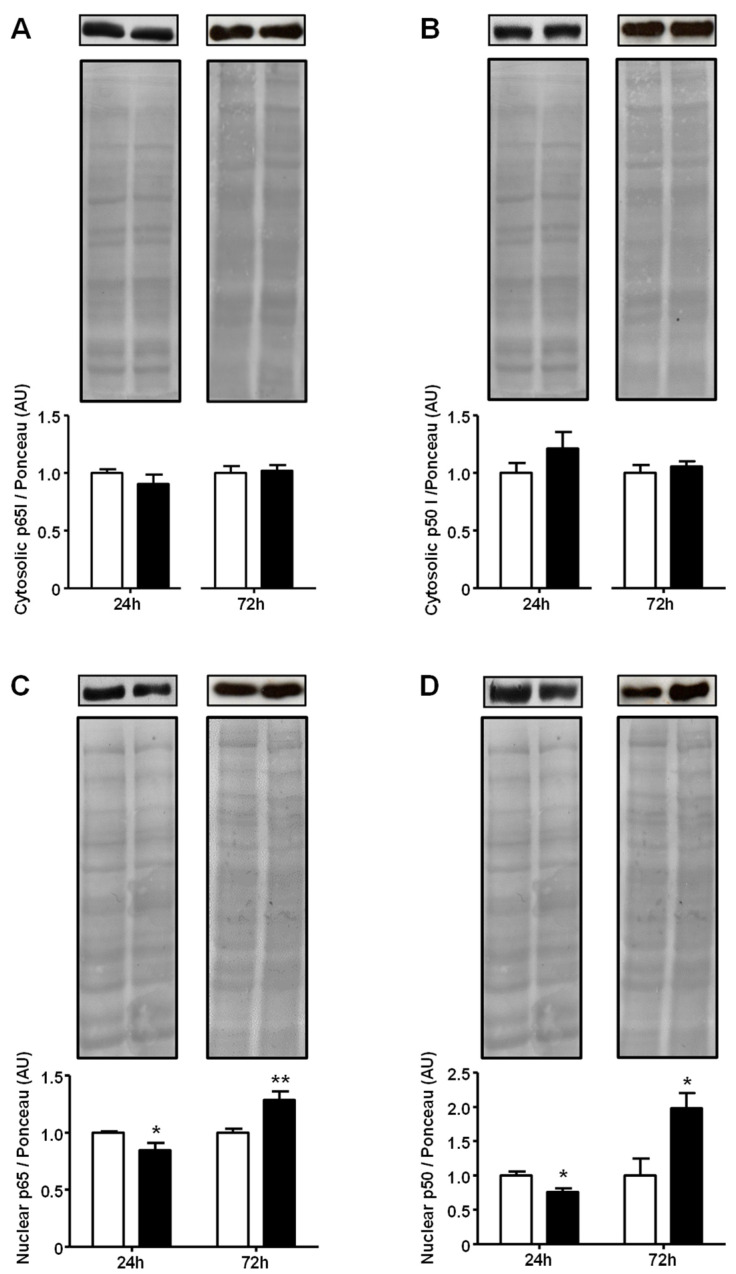
Advanced glycated albumin increases the nuclear content of nuclear factor NF-kappa-B at a 5.4 mg/dL concentration. Adipocytes were cultured for 24 h or 72 h in the presence of control albumin (CA; white bars) or advanced glycated albumin (GA; black bars) at a concentration of 5.4 mg/mL. NFKB/p65 (**A**,**C**) and NFKB/p50 (**B**,**D**) proteins were quantified by Western blotting, using the Ponceau-stained membrane as a loading control, in cytosolic (**A**,**B**) and nuclear (**C**,**D**) subcellular fractions. Representative blots of p65 and p50 are shown at the top of the panel with the respective Ponceau-stained membrane. Results were normalized considering the mean of CA values as 1.0 at each time-point. Data are expressed as the means ± SEM of 4 to 6 samples (from at least 4 different culture plates) and were compared using Student’s unpaired *t*-test (* *p* < 0.05 and ** *p* < 0.01 vs. CA). AU, arbitrary units; p50, nuclear factor NF-kappa-B/subunit 50; and p65: nuclear factor NF-kappa-B/subunit 65.

The nuclear localization of the NFKB protein subunits p65 and p50 is an important step in the signaling pathway. Thus, we examined the effect of high (5.4 mg/mL) GA concentration on the subcellular localization of these subunits. The cytosolic content of both p65 and p50 proteins showed no variation after either 24 h or 72 h of treatment. However, the nuclear contents of p65 and p50 subunits were clearly regulated in a time-related way. After 24 h, GA decreased p65 and p65 (*p* < 0.05); however, after 72 h, GA increased the nuclear content of p65 (30%, *p* < 0.01) and p50 (98%, *p* < 0.05) (Figure 4). Images of the original experiments from which the representative images were selected showing the entire Ponceau-stained membrane and the immunoblotted membrane are presented in Supplementary Figures S8–S11.

### 2.5. Advanced Glycated Albumin at 5.4 mg/mL Concentration Increases the Binding Activity of NFKB to the Slc2a4 Gene Promoter

**Figure 5:**
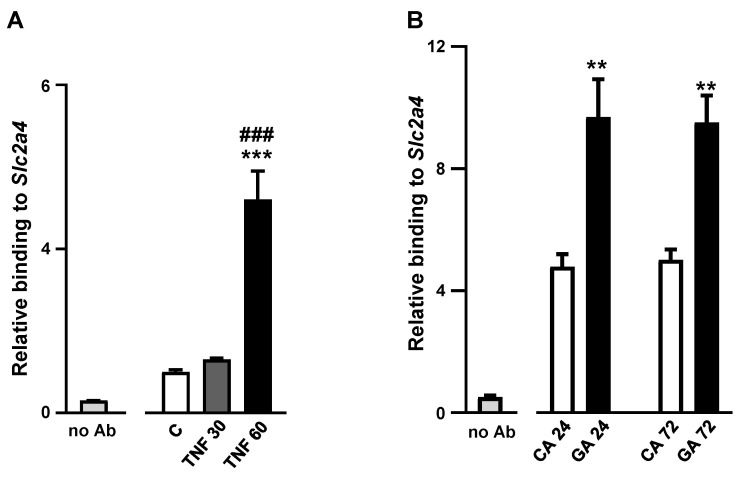
Advanced glycated albumin increases the binding activity of NFKB to the *Slc2a4* gene promoter. Adipocytes were cultured for 30 min (gray bar) or 60 min (black bar) with or without (white bar) 40 ng/mL tumor necrosis factor (TNF) (**A**) or for 24 h or 72 h with control albumin (CA, white bars) or advanced glycated albumin (GA, black bars) at a concentration of 5.4 mg/mL (**B**). After the indicated treatments, cells were processed for ChIP as described in the Methods section. The relative binding of p50 and p65 subunits to the *Slc2a4* promoter was quantified in relation to the total DNA (input); the unspecific binding of samples treated without an antibody (no Ab) is shown at the left of each panel. Data are the means ± SEM of 4 (**A**) or 4 to 5 (**B**) samples from three independent experiments and were compared by one-way ANOVA, followed by Tukey’s test (A: *** *p* < 0.001 vs. C; ### *p* < 0.001 vs. TNF 30; and B: ** *p* < 0.01 vs. CA 24 and CA 72). Ab, antibody anti-p50/p65; CA, control albumin; GA, advanced glycated albumin; TNF, tumor necrosis factor; and *Slc2a4*, solute carrier family 2 member 4).

Finally, to confirm that 5.4 mg/mL of GA can trigger NFKB participation in the repression of the *Slc2a4* gene, the p65/p50 binding activity at the NFKB binding site of the *Slc2a4* promoter was investigated. Firstly, we confirmed that in the present cellular system (3T3L1 adipocytes), TNF rapidly activates the NFKB pathway, achieving, within one hour, a more than five-fold increase (*p* < 0.001) in the p65/p50 relative binding activity (Figure 5A). Thus, the 5.4 mg/mL GA treatment was evaluated, and a two-fold increase (*p* < 0.01) in the p65/p50 binding activity was observed after both 24 h and 72 h of treatment (Figure 5B).

## 3. Discussion

The body’s AGE pool may increase under some pathophysiological conditions [3,6] as well as under excessive dietary AGE intake [5], generally participating in the genesis and/or aggravation of several disorders. Furthermore, in DM, AGEs generated by hyperglycemia contribute to aggravating insulin resistance, creating a vicious cycle [3]. In the present study, we reveal that advanced glycated-albumin (GA) impairs the expression of *Slc2a4*/GLUT4 in adipocytes in a dose- and time-modulated way and by a mechanism that ninvolves the activation of NFKB, which certainly contributes to the impairment of glycemic homeostasis.

Firstly, it is important to make it clear that this study was not designed to investigate the role of AGEs in adipogenesis, i.e., during the proliferation and differentiation of preadipocytes; instead, we analyzed the effects of AGEs upon already-differentiated adipocytes. Thus, the results described here do not contribute to explaining AGE’s effect on fat mass hyperplasia [22]; instead, they contribute to the induced metabolic imbalance of mature adipocytes, which can lead to the impairment of glycemic homeostasis by reducing cellular glucose uptake.

AGE-induced *Slc2a4*/GLUT4 regulation was considerably unique as, from the lowest (0.4 mg/mL) to the highest (5.4 mg/mL) concentrations of GA, the *Slc2a4* mRNA and GLUT4 protein were similarly regulated, increasing the expression at the lowest concentration after both 24 h and 72 h, and conversely decreasing the expression at the highest concentration after 72 h. The concentration-dependent regulation after 72 h particularly indicated a hormetic effect; although GA is considered a toxic compound, it displays a beneficial effect at low concentrations but a detrimental effect at high concentrations regarding the adipocyte capacity to express GLUT4 for glucose uptake. In addition, a hormetic effect may also be observed regarding the timeline since 5.4 mg/mL GA increases *Slc2a4*/GLUT4 expression after 24 h but decreases after 72 h. Similar results were described in differentiated 3T3L1 adipocytes exposed to 0.1 mg/mL GA, which revealed an increase in the total GLUT4 content for up to 12 h of exposition, but with a significant reduction after 48 h [23]; however, it is important to point out that the GA used in the latter study was much more glycated than the one used in our investigation [23]. Furthermore, in an investigation on the effect of exogenous AGE intake, methylglyoxal (MGO) challenged in gastrointestinal cells GIST-822 and Caco-2 and also revealed a hormetic effect regarding cell toxicity after 24 h, in which low concentrations increased, and high concentrations decreased cell viability compared to cells in the absence of MGO [24]. Finally, the hormetic effect of GA has already been proposed in response to the in vivo treatment of rats with GA, which has been shown to improve insulin sensitivity in the liver [25].

Still, regarding the deleterious repressor effect of GA on *Slc2a4*/GLUT4 observed here, it has already been described in the soleus muscle after a 2.5- to 7.5-h incubation with 1 mg/mL GA [17]. The GA-induced GLUT4 repression described here in adipocytes, along with their previous report in skeletal muscle, sheds light on how a high intake of dietary AGEs may impair glycemic homeostasis in humans, independently of a previous DM condition, thus contributing to the development of several pathophysiological conditions [12,13].

The present study also aimed to investigate the potential participation of inflammatory activity in *Slc2a4*/GLUT4 repression based on previous data that revealed the following: (1) the inflammatory-induced repression of *Slc2a4*/GLUT4, especially NFKB-mediated, has been extensively reported [3,26]; (2) NFKB (p65 and p50) binding to the *Slc2a4* promoter represses gene expression [26]; and (3) AGEs are widely known to stimulate inflammatory activity [3].

Firstly, we screened for the mRNA expression of *Nfkb1* and *Rela*, which are the key mediators of the inflammatory-induced repression of *Slc2a4*. An early 24-h increase in *Nfkb1* and *Rela* was observed in all GA concentrations. However, this activation was not sustained after 72 h, especially for *Rela*, indicating that some counter-regulatory response was triggered. Indeed, it is well-known that at an early phase of inflammatory activation, negative regulatory mechanisms are precociously set [27].

Since the GA-induced activation of *Nfkb1* and *Rela* gene expression was observed, the activation of the NFKB-mediated inflammatory pathway was investigated. These analyses were performed only at the highest GA concentration (5.4 mg/mL) because that concentration induced a harmful effect over a longer period (72 h) upon *Slc2a4*/GLUT4 expression. Firstly, in response to the GA treatment, the phosphorylation of IKKA/B (pIKKA and pIKKB) was shown to be unaltered after 24 h and 72 h; however, the nuclear content of NFKB-p50/p65 was greatly modulated. Despite the initial transient decrease at 24 h in GA incubation, the NFKB-p50/065 nuclear content rapidly increased at 72 h of treatment independently of the unchanged cytosolic content. These results indicate that the upstream step in the canonical NFKB pathway (IKKA/B phosphorylation) was not activated at the evaluated times; however, the downstream step (p50/p65 nuclear translocation) was greatly activated after 72 h.

In the canonical pathway of NFKB signaling, IKKA/B phosphorylation leads to IKBA (NF-kappa-B inhibitor alpha) phosphorylation, with subsequent ubiquitination followed by proteasomal degradation; thus, p50/p65 complexes are released to translocate to the nucleus and regulate the gene expression of target genes [28]. Considering that, under the present experimental condition, GA did not activate the canonical pathway of NFKB, it did activate the nuclear translocation of p50/p65. Furthermore, this NFKB activation should not be considered a noncanonical pathway, which, in most circumstances, consists of the p52/RelB activation [28]; thus, this regulation should instead be considered as an atypical pathway of NFKB activation involving IKK-independent mechanisms of p50/p65 nuclear localization and DNA binding, which is a mechanism that has been reported for decades [28,29,30].

Still, regarding the above, we have already described in L6 muscle cells that the palmitate-induced repression of *Slc2a4* gene expression involves increased NFKB nuclear content and binding to the *Slc2a4* promoter despite unchanged IKKB/A activation [3]. Furthermore, the 12-week-GA-treatment-induced repression of *Slc2a4* gene expression in the soleus muscle also involved increased nuclear p50/p65 content despite unchanged IKKA/B activation [17]. On the other hand, in vitro, 2.5-h GA incubation of the soleus muscle from healthy rats also increased nuclear p50/p65 content, but in this case, showed increased IKKA/B phosphorylation and reduced content of IKBA/B [17]. All in all, we can suppose that the present results and others from the literature indicate an NFKB activation that (1) involves an IKKA/B activation, which could be transient, making it difficult to detect the exact time it occurs, or (2) involves an atypical pathway of IKK-independent NFKB activation.

Finally, the last step of the activation of the NFKB pathway related to the inhibition of *Slc2a4* gene expression is the binding of p50/p65 to the NF-κB binding site at the *Slc2a4* promoter [26]. In the present study, after confirming the classic TNF-induced increase in p50/p65 binding to the *Slc2a4* gene promoter, the results revealed that after 24 h and 72 h of GA treatment of adipocytes, p50/p65 binding to the target gene increased, finally demonstrating that the GA inhibition of *Slc2a4*/GLUT4 expression involves the activation of NFKB-mediated inflammatory pathway.

Interestingly, the effects of AGEs have been seen to be maintained after their initiation, which might explain the AGE-induced (5.4 mg/dL, 72 h) increase in NFKB activity when *Rela* and *Nfkb1* gene expressions no longer increased. GA, drawn from subjects with uncontrolled DM or produced in vitro (as in the present investigation), can sensitize macrophages to inflammation induced by lipopolysaccharide. This effect persists for extended periods, even after the removal of GA from the cell media, impacting cell metabolism [3], and relies on the sustained nuclear activation of NF-KB-p65 [31].

In DM, several dysfunctions have been associated with AGE-induced cellular injuries, participating in various diabetes-related degenerative diseases, and most of them have been confirmed to induce cellular damage in vitro [3,11]. In vitro, the GA-induced repression of *Slc2a4*/GLUT4 expression was first reported in muscle, but now it is described to occur in adipocytes, the effects of which can lead to participation in insulin resistance and thus contribute to inducing DM and/or worsening pre-existing DM. Still, regarding the GA-induced reduction in GLUT4 expression, it has been recently reported in SH-SY5Y human neurons and also accompanied by the increased binding activity of p50/p65 to the *Slc2a4* promoter; in addition, in this neuron, TNF treatment impairs the expression of several markers of neuronal function, revealing an inflammatory-mediated GA-induced neuronal dysfunction [20]. Moreover, in postmortem brains from DM subjects, a reduction in GLUT4 expression and the neuronal soma area was observed in hippocampal neurons, together with increased expression of NFKB-p65 [20]. These results place the reduction in GLUT4 expression as an important player not only in the peripheral impairment of glucose homeostasis but also in the development of DM-related cognitive dysfunction.

## 4. Materials and Methods

### 4.1. Modification of Bovine Albumin by Advanced Glycation In Vitro

Bovine fatty acid-free albumin (40 mg; Sigma-Aldrich, Steinheim, Germany) was incubated with freshly prepared 10 mM glycolaldehyde (GAD; Sigma-Aldrich; Fluka-Buchs, Germany) and dissolved in PBS with EDTA (pH = 7.4), for 4 days at 37 °C, under sterile conditions, a nitrogen atmosphere, in the dark and in a shaking bath water. Control albumin (CA) was incubated with phosphate buffer only under the same conditions. After extensive dialysis, samples were sterilized through a 0.22 µm filter, and the protein concentration was determined using the Lowry technique [32]. Endotoxin levels in the albumin samples were below 50 pg/mL (Limulus Amebocyte Lysate (LAL) assay; Cape Cod, Falmouth, MA, USA) [17].

### 4.2. Cell Culture and Treatments

Mouse 3T3-L1 preadipocytes were obtained from the American Type Culture Collection, Rio de Janeiro Cell Bank, Rio de Janeiro, Brazil (ATCC^®^ Number: CL-173TM) and cultured as previously described [26]. Briefly, the cells were induced to differentiate using DMEM (Dulbecco’s modified Eagle medium, Vitrocell Embriolife, Campinas, Brazil), 10% FBS (fetal bovine serum; Vitrocell Embriolife, Campinas, SP, Brazil), supplemented with 10 μg/mL regular insulin, 1 μM dexamethasone, and 0.5 mM 3-isobutyl-1-methylxanthine (Sigma-Aldrich; St. Louis, MO, USA) for 6 days, followed by 2 days without supplementation. Differentiation was completed on day 8, and after that, the cells were transferred to a DMEM with 5.5 mM glucose, 10% FBS, 1% antibiotics (penicillin/streptomycin), and 1.5 nM insulin. Thus, treatments were initiated with bovine control albumin (CA) or advanced glycated albumin (GA), obtained as previously described [17,33] at concentrations of 0.4, 3.6, or 5.4 mg/mL, for 24 or 72 h, as specified in the Results. Additional experiments were performed in cells treated or untreated with tumor necrosis factor (TNF) (Sigma-Aldrich, #H8916) at 40 ng/mL [33] for 30 min or 60 min. Adipocyte differentiation was confirmed by staining a set of culture plates with oil red O, and in all experimental protocols, a reduction in yellow tetrazolium salt 3-(4,5-dimethylthiazol-2-yl)-2,5-diphenyltetrazolium bromide (MTT) was used to evaluate cellular viability as previously described [33].

### 4.3. Reverse Transcription and Quantitative Polymerase Chain Reaction (RT-qPCR)

For mRNA gene expression analysis, total RNA from adipocytes was extracted using TRIzol^®^ Reagent according to manufacturer recommendations (Invitrogen, Carlsbad, CA, USA), and the reverse transcriptase reaction was performed using random primers (Invitrogen, Carlsbad, CA, USA), and the ImProm-II^®^ Reverse Transcription System (Promega Corporation, Madison, WI, USA). Quantitative PCR amplification was performed using the PowerUp^®^ SYBR^®^ Green Master Mix (Applied Biosystems Inc., Foster City, CA, USA) and a StepOne Plus Instrument (Applied Biosystems Inc.); inventoried probes (Applied Biosystems) were used for mouse *Slc2a4* (Mm01245502_m1), mouse *nuclear factor kappa B subunit 1* (*Nfkb1*) and *RELA proto-oncogene* (*Rela*) genes (Mm00476363_m1 and Mm00501346_m1, respectively). Relative expression values of the different genes were calculated from the threshold cycle (Ct) following the 2^–ΔΔCt^ method; *Gapdh* (Mm99999915_g1) was chosen as a reference gene after previous RefFinder algorithm analysis testing of *Gapdh*, *Atp5b*, and *Actb* genes. Results were expressed as arbitrary units (AUs) related to the mean of the controls, which was set to 1.0.

### 4.4. Western Blotting

For protein expression analyses, adipocytes were processed to obtain different subcellular protein fractions according to the target protein. GLUT4, and the phosphorylated inhibitor of nuclear factor kappa B-kinase subunits alpha (pIKKA) and beta (pIKKB) proteins were measured in a total cellular protein fraction [17]. Nuclear factor NF-kappa B (NFKB) subunits p50 and p65 were measured in both cytosolic and nuclear protein fractions, obtained as previously described [17,33]. The total protein content in the samples was quantified using the Bradford method according to manufacturer recommendations (Bio-Rad Laboratories; Hercules, CA, USA). Equal amounts of protein were electrophoresed, transferred into the nitrocellulose membrane, and stained with Ponceau for further normalization of the results. Membranes were immunoblotted with anti-GLUT4 (Milipore, Burlington, MA, USA, #071404, 1:3000), anti-p65 (Abcam, Cambridge, UK, #ab-7970, 1:1000), anti-p50 (Cell Signaling, Danvers, MA, USA, #12540, 1:1000), or anti-pIKKA/B (Santa Cruz, Dallas, TX, USA, #sc-23470-R, 1:1000) antibodies, followed by appropriate secondary conjugated antibodies. The enhanced chemiluminescence (ECL) procedure was performed using the SuperSignal^®^ West Pico Plus Chemiluminescent Substrate (Thermo Scientific; Rockford, IL, USA). The images were taken using the Syngene automated system model G:BOX Chemi XRQ (Synoptics, CA, Cambridge, UK) and the optical density of the blots was analyzed using Image J software (National Institutes of Health, Bethesda, MD, USA version 1.51). The densities of the respective lanes from the Ponceau-stained membrane were used for normalization. The results were expressed as arbitrary units (AUs) related to the mean of the controls, which was set to 1.0.

### 4.5. Chromatin Immunoprecipitation (ChIP) Assay

After treatments with TNF, CA, or GA, as described above, the cells were treated with formaldehyde, transferred to a lysis buffer, and DNA shearing was performed with sonication to obtain fragments of 200 to 1000 bp. Equal amounts of the samples were diluted in a dilution buffer containing protein A-sepharose saturated with salmon sperm DNA, incubated at 4 °C for 4 h, and centrifuged at 3000× *g* for 1 min. The supernatants were recovered, aliquots (10 µL) were collected as “input”, and the remaining supernatants were incubated with the 5 µg anti-p65 antibody (Abcam #ab7970) and were thus subjected to immunoprecipitation with protein A-sepharose saturated with salmon sperm DNA. In parallel, samples were incubated without antibody (no-Ab) to generate negative controls. Sepharose pellets were washed, treated with elution buffer, and centrifuged (3000× *g* for 1 min). Supernatants were subjected to crosslinking reversal and RNase A treatment. DNA was purified with phenol-chloroform and resuspended in H_2_O. DNA samples were used to amplify the −208/+2 segment of the *Slc2a4* promoter, containing the binding sites of NFKB [26], and using the primers 5′–TGAAAACTCAGAAGCAGGCG–3′ (sense) and 5′–GCTCTCCGGGATCTAGTGAG–3′ (anti-sense). The products were amplified using qPCR (Platinum^®^ SYBR^®^ Green qPCR SuperMix UDG, Invitrogen Life Technologies, Carlsbad, CA, USA), and the input value from each sample was used as a control for the amount of chromatin used in the ChIP reaction. The 2^−ΔΔCT^ formula was used to calculate the relative binding of NFKB (p50/p65) to the *Slc2a4* promoter.

### 4.6. Statistical Analysis

Data were expressed as means ± standard errors of the mean (SEM). A comparison between the two groups was performed using an unpaired Student’s *t*-test. Comparisons among 3 or 4 groups were performed by one-way analysis of variance (ANOVA), followed by Tukey’s test, after confirming the normality of the data distribution with the Shapiro–Wilk test. For each concentration of CA and GA, cellular viability at different times (24 h or 72 h) was analyzed by one-way ANOVA. To depict the effects of time and/or treatment, the GLUT4 protein was additionally analyzed as follows: as the results of different concentrations of CA showed no variation within each period (24 h or 72 h), these data were pooled into two groups (24 h CA and 72 h CA); thus, all values were normalized by considering the mean of 24 h CA as 1.0 and analyzed by two-way ANOVA to verify the effect of time, treatment, and interaction; afterward, the mean of each GA concentration was compared with their respective CA group (at each time-point) by Student’s unpaired *t*-test; comparisons were considered statistically significant at *p* < 0.05. Analyses were performed using GraphPad Prism 10.

## 5. Conclusions

This study shows that the 72-h exposure of mature adipocytes to advanced glycated albumin triggers a proinflammatory response, which involves the activation of the atypical NFKB pathway, ultimately leading to the repression of the *Slc2a4* gene and its GLUT4 protein, which can compromise adipocyte glucose utilization. This reveals that AGEs may not only worsen the glycemic control of DM subjects but also impair the glycemic homeostasis of non-DM subjects under high intake of AGE-rich foods.

## Data Availability

The original contributions presented in the study are included in the article/supplementary material, further inquiries can be directed to the corresponding author.

## Supplementary Materials

The supporting information can be downloaded at: https://www.mdpi.com/article/10.3390/ijms25158242/s1.

## Abbreviations

AGEs	advanced glycation end products
AU	arbitrary units
CA	control albumin
ChIP	chromatin immunoprecipitation
CA	control albumin
DM	diabetes mellitus
DMEM	Dulbecco’s modified Eagle medium
FBS	fetal bovine serum
GA	advanced glycated albumin
*Gapdh*	glyceraldehyde-3-phosphate dehydrogenase (mouse gene)
GLUT4	solute carrier family 2, facilitated glucose transporter member 4
IKBA	NF-kappa-B inhibitor alpha
IKKA	inhibitor of nuclear factor kappa-B kinase subunit alpha
IKKB	inhibitor of nuclear factor kappa-B kinase subunit beta
MGO	methylglyoxal
MTT	tetrazolium salt 3-(4,5-dimethylthiazol-2-yl)-2,5-diphenyltetrazolium bromide
NFKB	nuclear factor NF-kappa-B
NFKB/p50	nuclear factor NF-kappa-B/subunit 50
NFKB	nuclear factor NF-kappa-B/subunit 65
*Nfkb1*	nuclear factor kappa B subunit 1 (mouse gene) codifies the p50 subunit
p50/p65	NFKB/p50 and NFKB/p65 complex
*Rela*	RELA proto-oncogene, NF-kB subunit (mouse gene) codifies the p65 subunit
RT-qPCR	reverse transcription and quantitative polymerase chain reaction
*Slc2a4*	solute carrier family 2 member 4 (mouse gene)
*SLC2A4*	solute carrier family 2 member 4 (human gene)
TNF	tumor necrosis factor
