# Supplementary material for: Nuclear Factor-Kappa-B Mediates the Advanced Glycation End Product-Induced Repression of Slc2a4 Gene Expression in 3T3-L1 Adipocytes
Source: Int J Mol Sci. 2024 Jul 28;25(15):8242. doi: 10.3390/ijms25158242 (PMC11311564; doi:10.3390/ijms25158242)
Supplement: Supplementary file 1 [file ijms-25-08242-s001.zip › ijms3105422Supplementary Figures [R].pptx]

## Slide 1
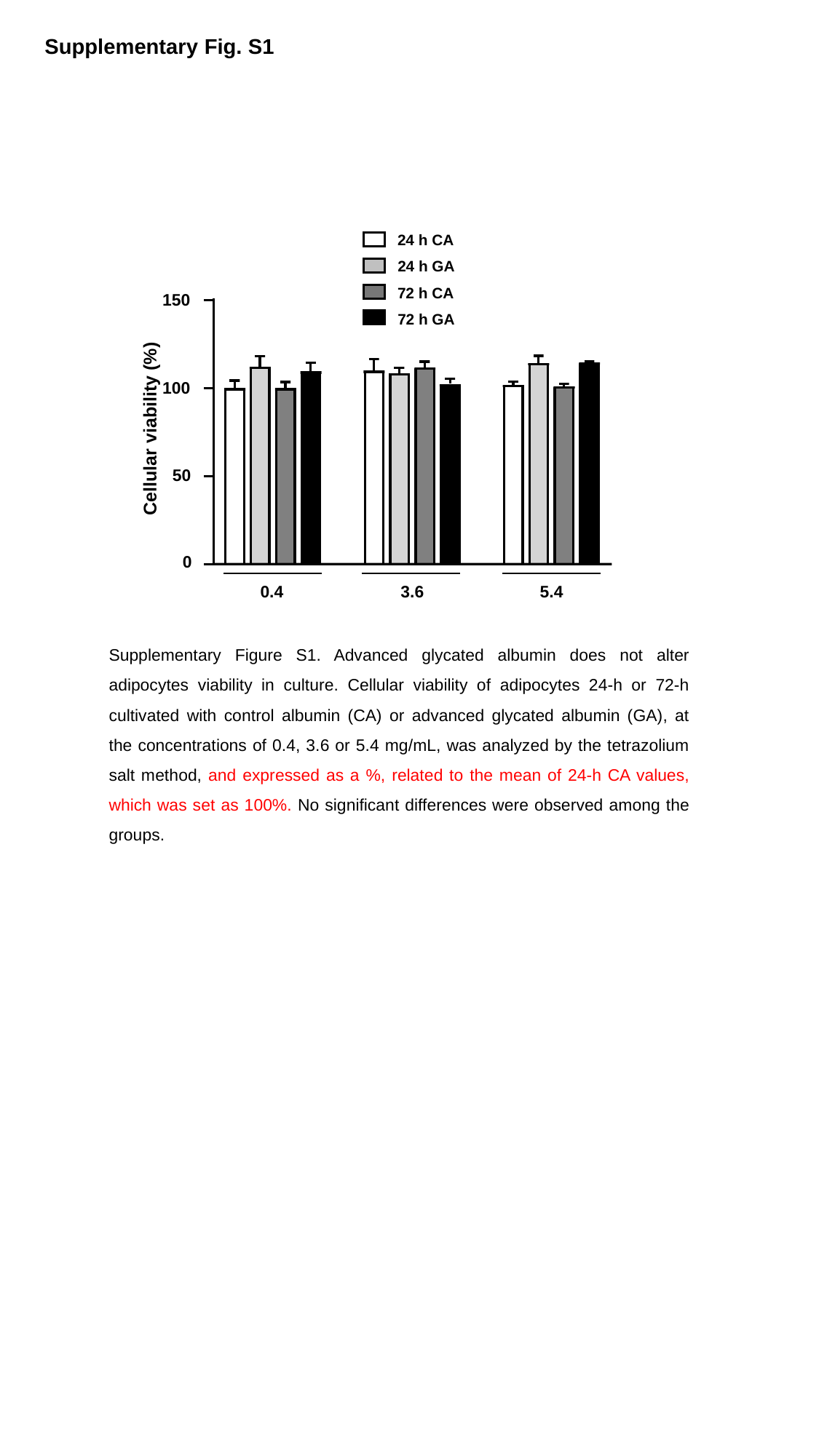

Supplementary Fig. S1
24 h CA
24 h GA
72 h CA
72 h GA
150
100
Cellular viability (%)
50
0
0.4
3.6
5.4
Supplementary Figure S1. Advanced glycated albumin does not alter adipocytes viability in culture. Cellular viability of adipocytes 24-h or 72-h cultivated with control albumin (CA) or advanced glycated albumin (GA), at the concentrations of 0.4, 3.6 or 5.4 mg/mL, was analyzed by the tetrazolium salt method, and expressed as a %, related to the mean of 24-h CA values, which was set as 100%. No significant differences were observed among the groups.

## Slide 2
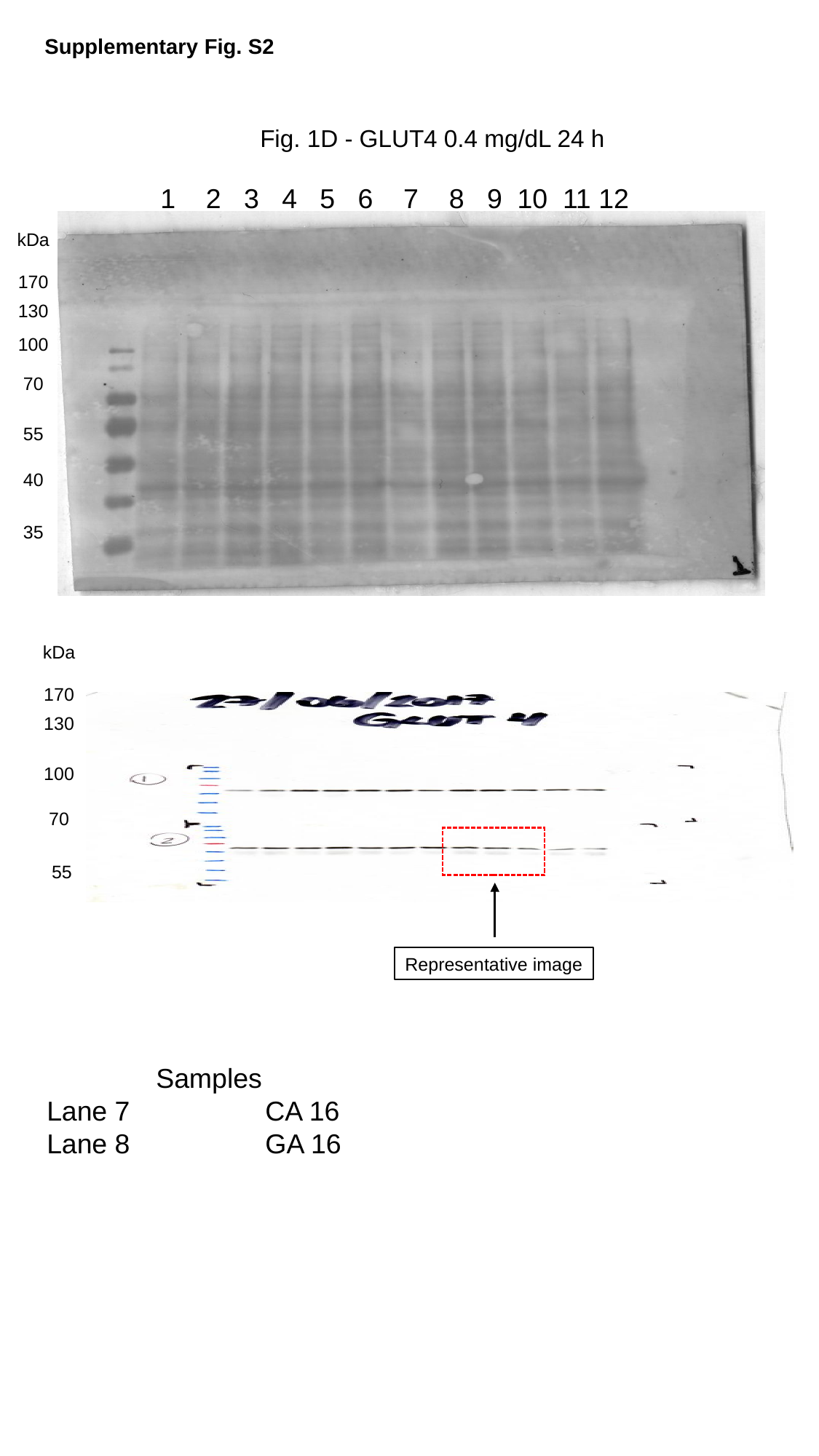

Supplementary Fig. S2
Fig. 1D - GLUT4 0.4 mg/dL 24 h
1 2 3 4 5 6 7 8 9 10 11 12
kDa
170
130
100
70
55
40
35
kDa
170
130
100
70
55
Representative image
			Samples
Lane 7		CA 16
Lane 8		GA 16

## Slide 3
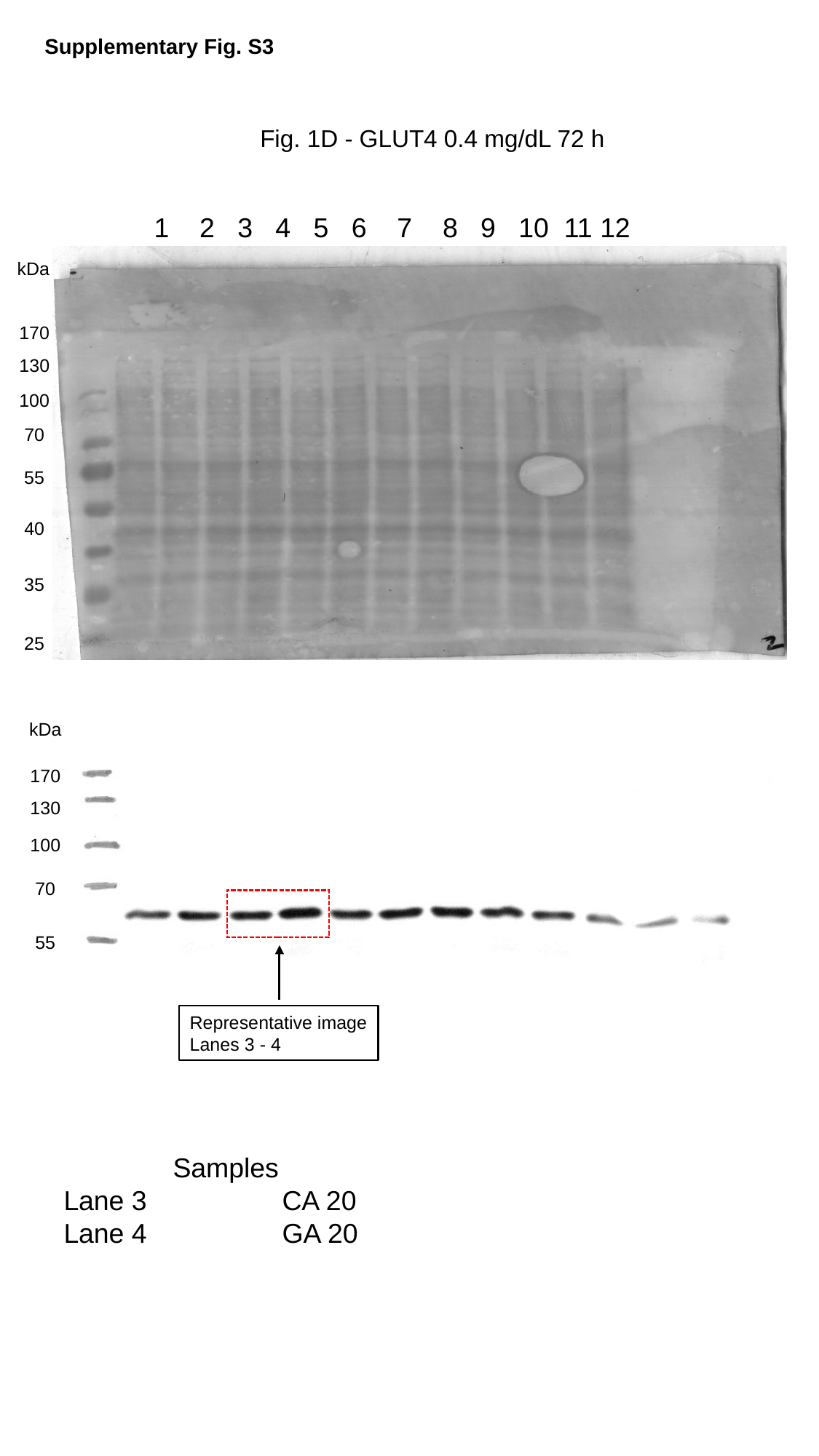

Supplementary Fig. S3
Fig. 1D - GLUT4 0.4 mg/dL 72 h
1 2 3 4 5 6 7 8 9 10 11 12
kDa
170
130
100
70
55
40
35
25
kDa
170
130
100
70
55
Representative image
Lanes 3 - 4
			Samples
Lane 3		CA 20
Lane 4		GA 20

## Slide 4
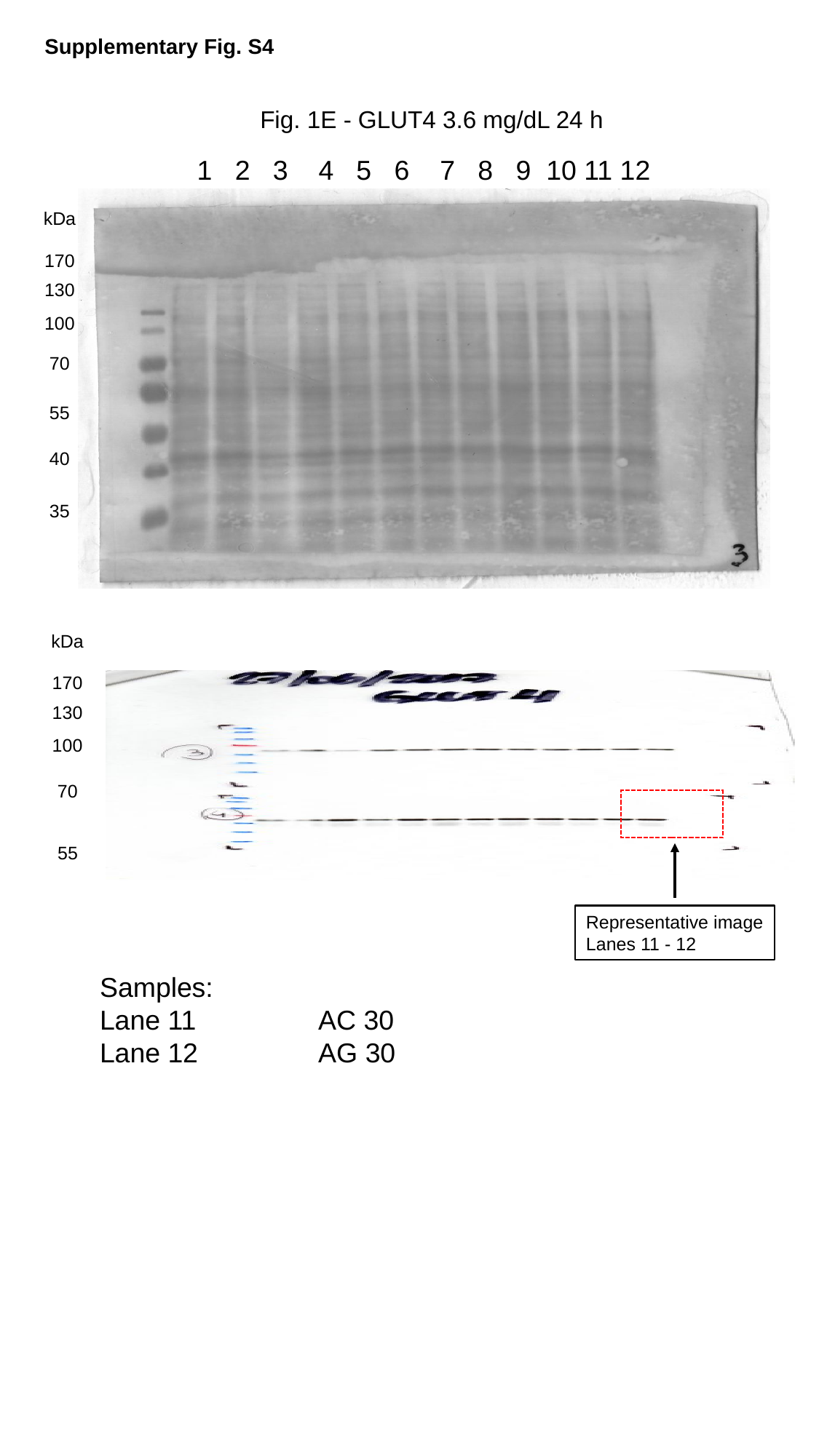

Supplementary Fig. S4
Fig. 1E - GLUT4 3.6 mg/dL 24 h
1 2 3 4 5 6 7 8 9 10 11 12
kDa
170
130
100
70
55
40
35
kDa
170
130
100
70
55
Representative image
Lanes 11 - 12
			Samples:
Lane 11		AC 30
Lane 12		AG 30

## Slide 5
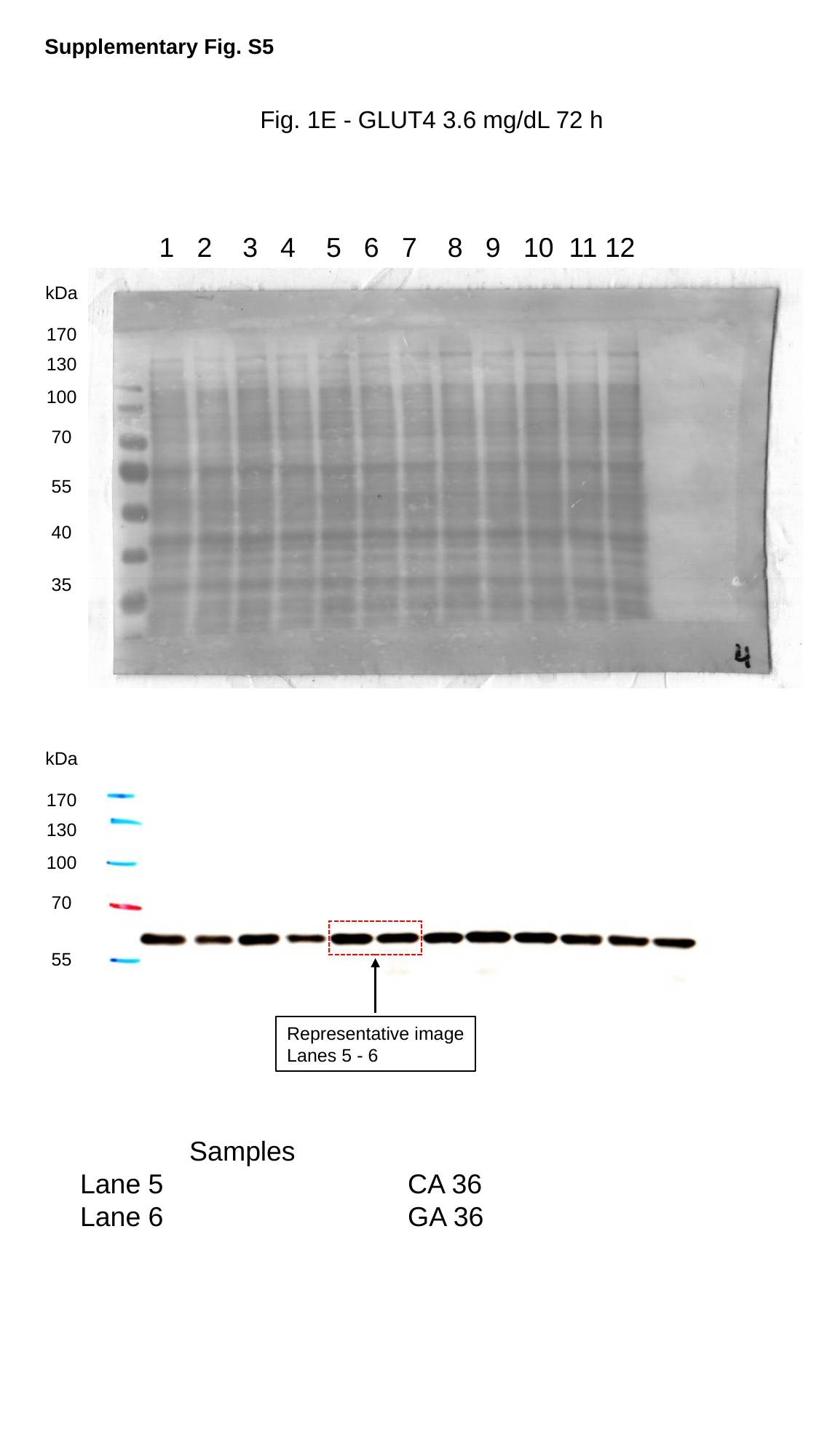

Supplementary Fig. S5
Fig. 1E - GLUT4 3.6 mg/dL 72 h
1 2 3 4 5 6 7 8 9 10 11 12
kDa
170
130
100
70
55
40
35
kDa
170
130
100
70
55
Representative image
Lanes 5 - 6
				Samples
Lane 5			CA 36
Lane 6			GA 36

## Slide 6
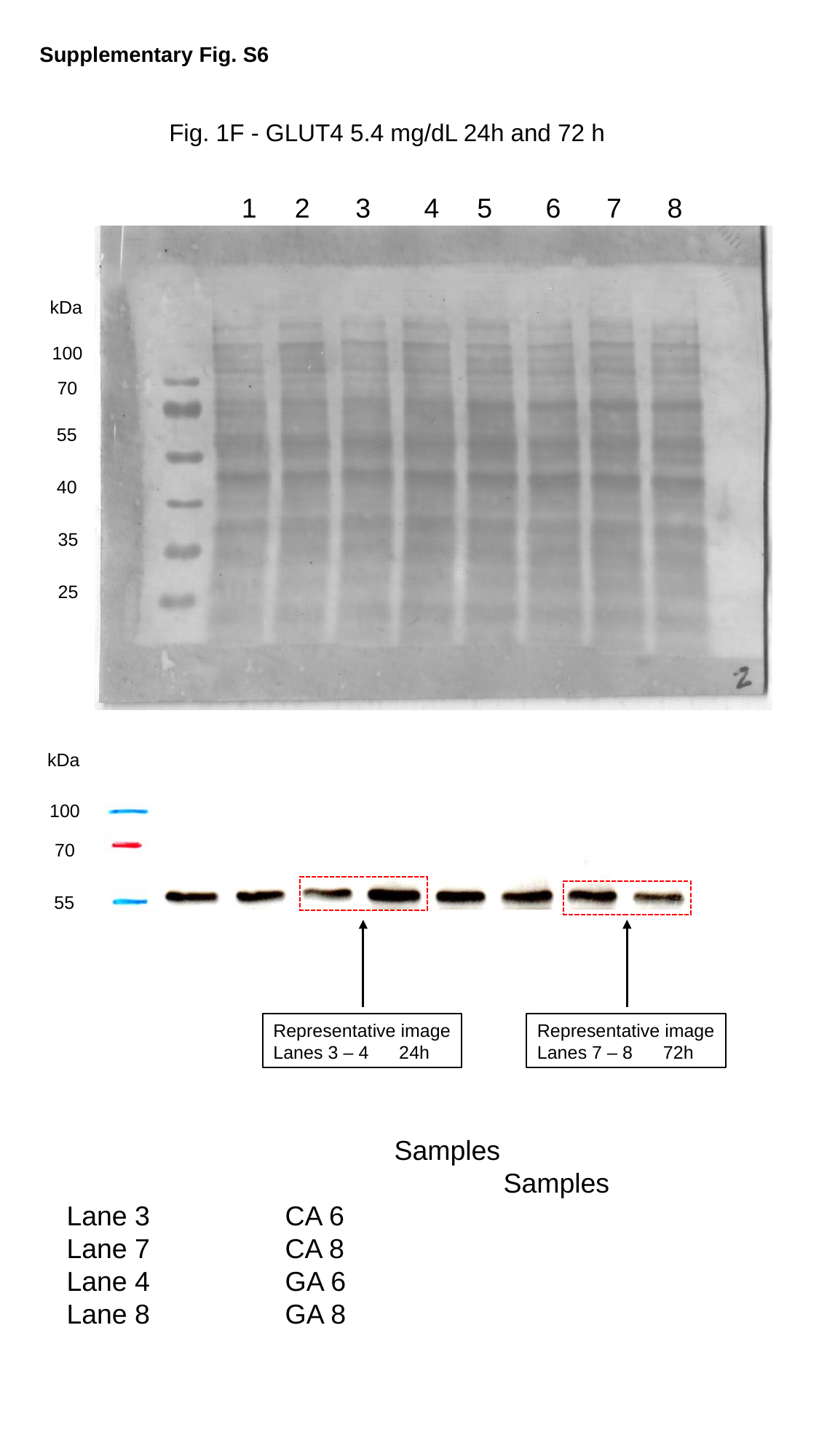

Supplementary Fig. S6
Fig. 1F - GLUT4 5.4 mg/dL 24h and 72 h
1 2 3 4 5 6 7 8
kDa
100
70
55
40
35
25
kDa
100
70
55
Representative image
Lanes 7 – 8 72h
Representative image
Lanes 3 – 4 24h
			Samples							Samples
Lane 3		CA 6				Lane 7		CA 8
Lane 4		GA 6				Lane 8		GA 8

## Slide 7
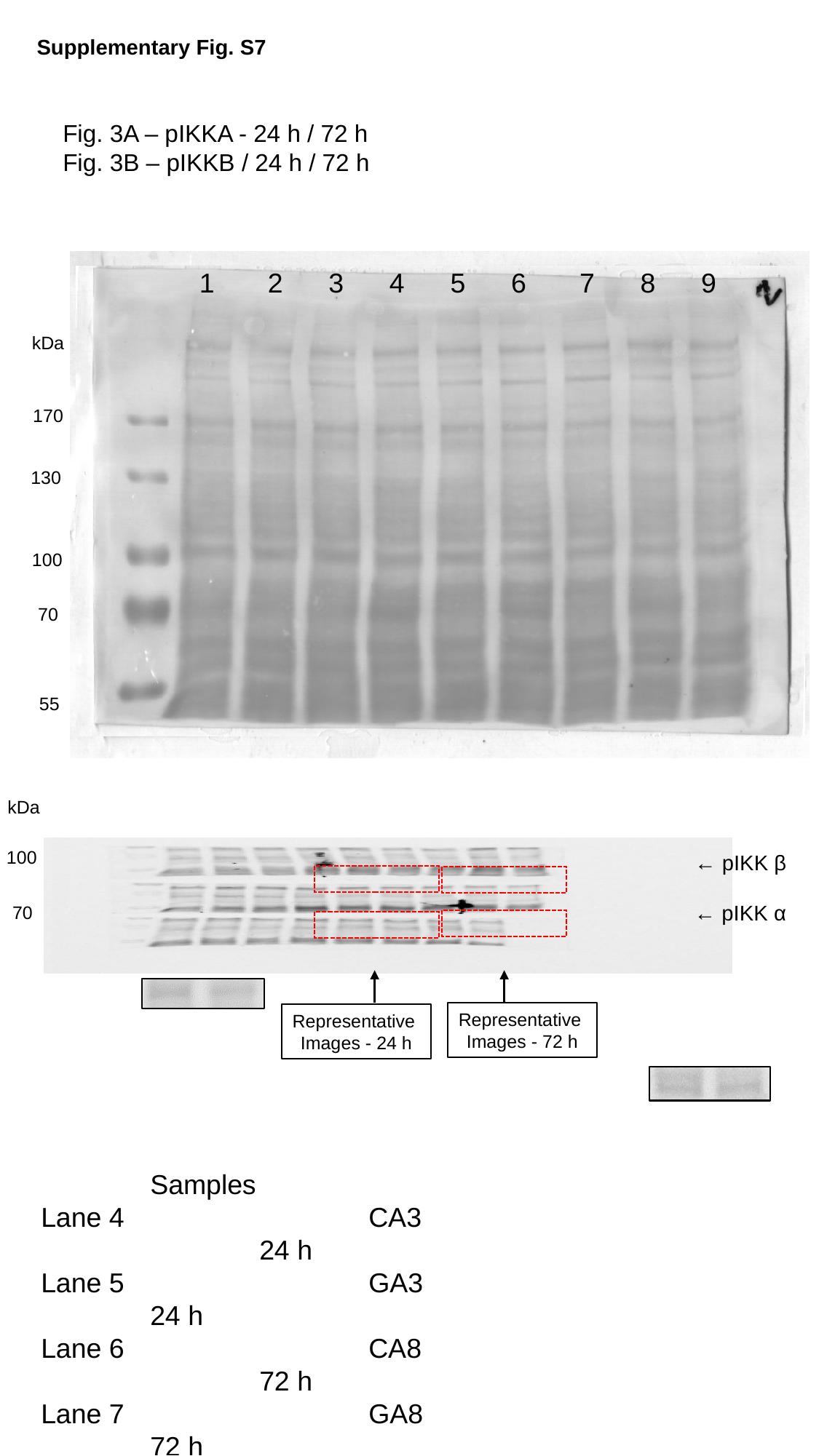

Supplementary Fig. S7
Fig. 3A – pIKKA - 24 h / 72 h
Fig. 3B – pIKKB / 24 h / 72 h
1 2 3 4 5 6 7 8 9
kDa
100
70
55
170
130
kDa
100
70
← pIKK β
← pIKK α
Representative
Images - 24 h
Representative
Images - 72 h
				Samples
Lane 4			CA3		24 h
Lane 5			GA3 	24 h
Lane 6			CA8		72 h
Lane 7			GA8	72 h

## Slide 8
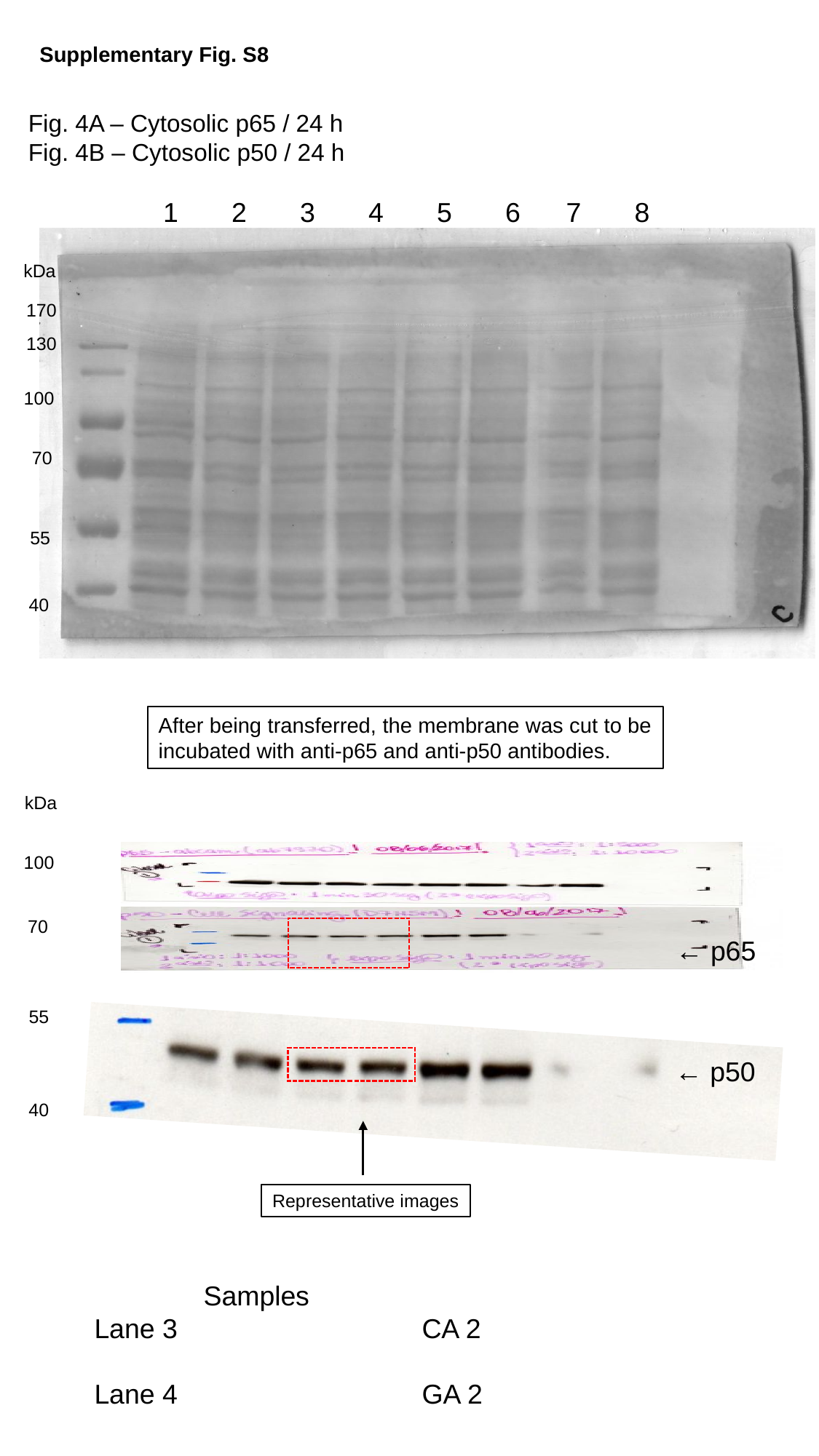

Supplementary Fig. S8
Fig. 4A – Cytosolic p65 / 24 h
Fig. 4B – Cytosolic p50 / 24 h
1 2 3 4 5 6 7 8
kDa
100
70
55
40
170
130
After being transferred, the membrane was cut to be
incubated with anti-p65 and anti-p50 antibodies.
kDa
100
70
55
40
← p65
← p50
Representative images
				Samples
Lane 3			CA 2
Lane 4			GA 2

## Slide 9
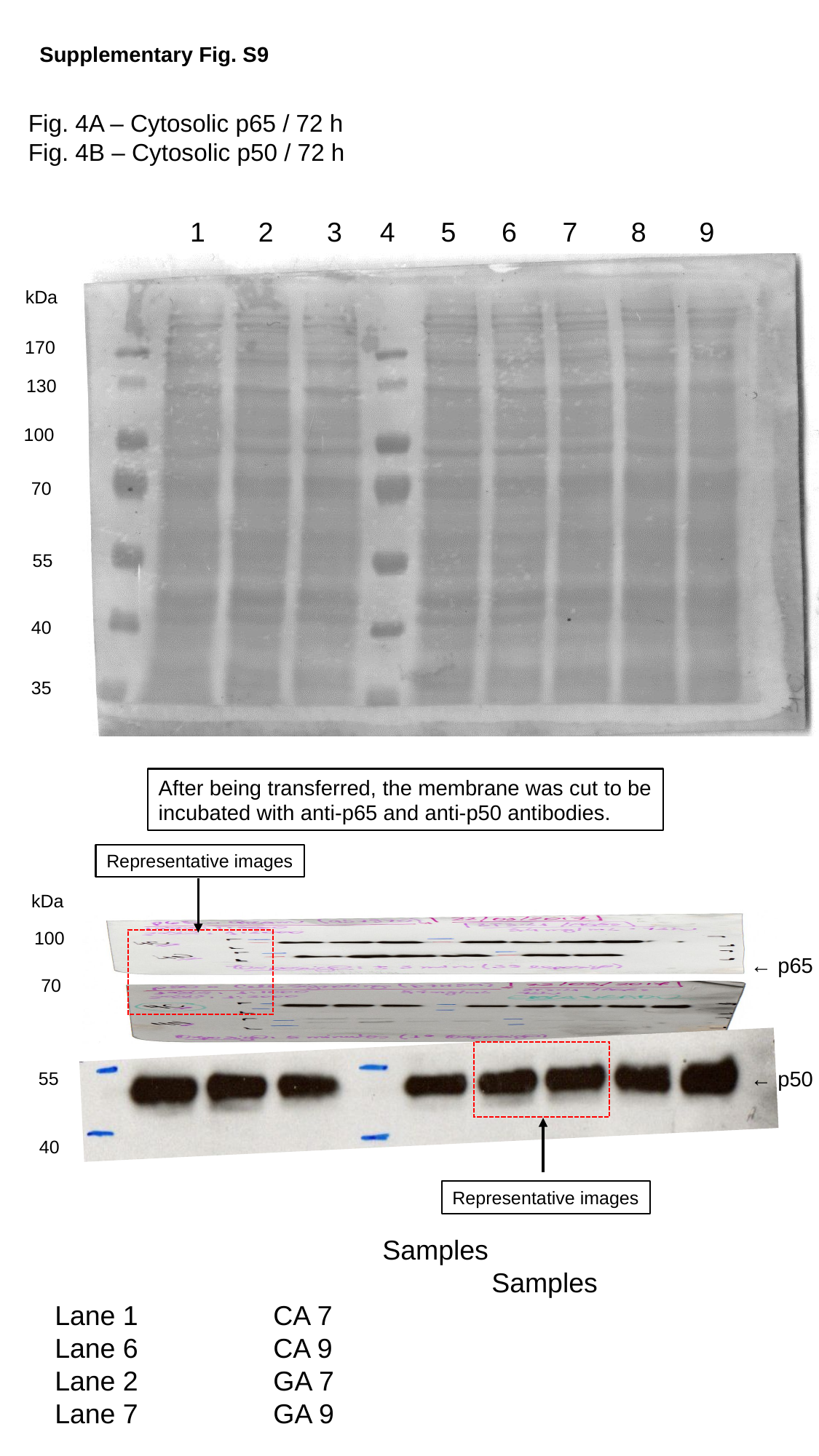

Supplementary Fig. S9
Fig. 4A – Cytosolic p65 / 72 h
Fig. 4B – Cytosolic p50 / 72 h
1 2 3 4 5 6 7 8 9
kDa
100
70
55
40
170
130
35
After being transferred, the membrane was cut to be
incubated with anti-p65 and anti-p50 antibodies.
Representative images
kDa
100
70
55
40
← p65
← p50
Representative images
			Samples							Samples
Lane 1		CA 7				Lane 6		CA 9
Lane 2		GA 7				Lane 7		GA 9

## Slide 10
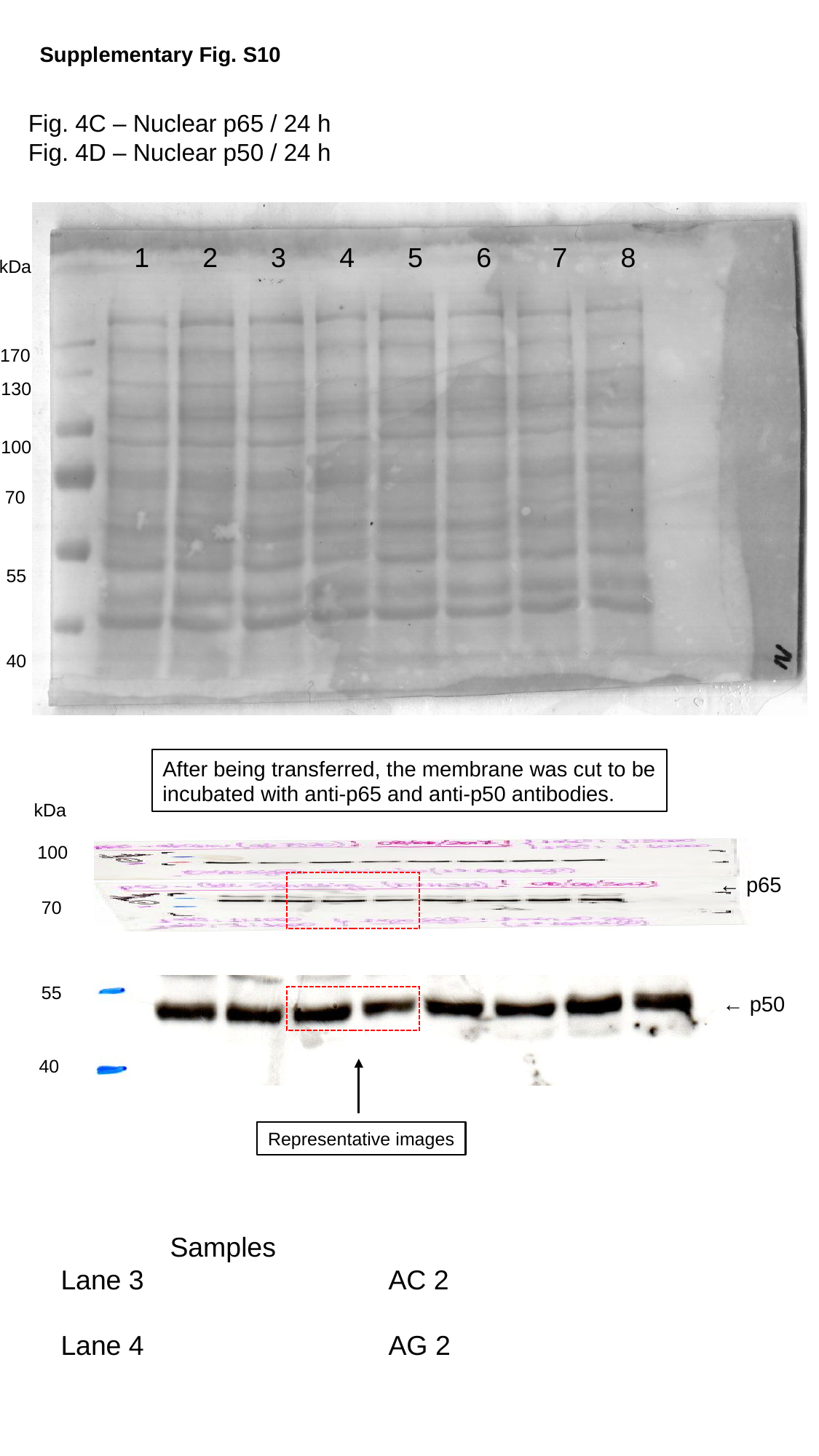

Supplementary Fig. S10
Fig. 4C – Nuclear p65 / 24 h
Fig. 4D – Nuclear p50 / 24 h
1 2 3 4 5 6 7 8
kDa
100
70
55
40
170
130
After being transferred, the membrane was cut to be
incubated with anti-p65 and anti-p50 antibodies.
kDa
100
70
55
40
← p65
← p50
Representative images
				Samples
Lane 3			AC 2
Lane 4			AG 2

## Slide 11
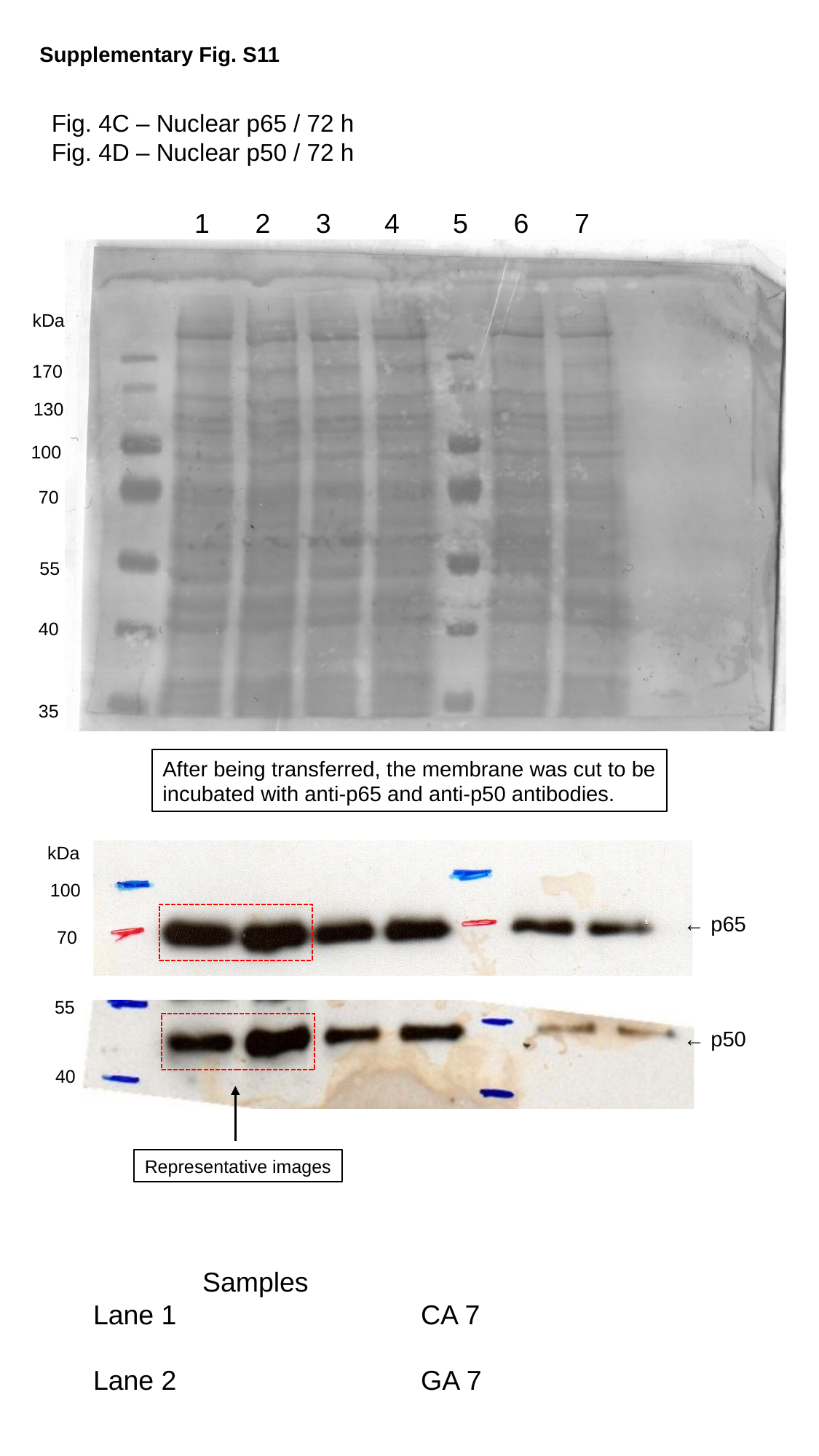

Supplementary Fig. S11
Fig. 4C – Nuclear p65 / 72 h
Fig. 4D – Nuclear p50 / 72 h
1 2 3 4 5 6 7
kDa
100
70
55
40
170
130
35
After being transferred, the membrane was cut to be
incubated with anti-p65 and anti-p50 antibodies.
kDa
100
70
55
40
← p65
← p50
Representative images
				Samples
Lane 1			CA 7
Lane 2			GA 7
